# Supplementary material for: Acceptability of decentralizing childhood tuberculosis diagnosis in low-income countries with high tuberculosis incidence: Experiences and perceptions from health care workers in Sub-Saharan Africa and South-East Asia
Source: PLOS Glob Public Health. 2023 Oct 11;3(10):e0001525. doi: 10.1371/journal.pgph.0001525 (PMC10566691; doi:10.1371/journal.pgph.0001525)
Supplement: S1 File — (DOCX) [file pgph.0001525.s001.docx]

**Acceptability of decentralizing childhood tuberculosis diagnosis in low-income countries with high tuberculosis incidence: experiences and perceptions from health care workers in sub-Saharan Africa and South-East Asia**

**Supplementary Material**

S1 File. **Individual interview guide for Health care workers, TB-Speed Decentralization study (2018-22).**

# Systematic screening for paediatric TB in outpatients/at triage

# Can you share your experience of implementing systematic screening as part of TB Speed project? What is working well and what is not working well? And WHY so?

# *Probe for example for:*

# *Systematic screening or not? Why not?*

# *By whom ? Which entry points?*

# *How did staff re-organise their tasks to accommodate systematic screening? Change in patient flow as compared to prior TB-Speed?*

# *Did it occur sometimes that screening could not take place because of lack of trained staff onsite or because of other priorities?*

# *Parents’ understanding / attitude towards screening procedures and questions? Towards referral process if required?*

# *Effect of COVID pandemic in screening*

# *Personal experience and feeling towards screening children for TB in this facility ETC…*

# *How did it change overall practices, or overall understanding/perceptions of childhood TB.*

# Can you share your experience whether or not systematic screening contributed to find presumptive TB cases in your facility? And WHY?

# *Probe for example for:*

# *Quality of the screening process? reliable? good strategy? (good use of time/money?)*

# What would you suggest to improve systematic screening of children for TB in this facility?

# Would you recommend systematic screening as routine in your facility? And WHY?

# Naso-Pharyngeal Aspirate (NPA)

# Can you share your experience of NPA as part of TB Speed project? What is working well and what is not working well? And WHY so?

# *Probe for example for:*

# *Personal involvement in NPA*

# *Any technical difficulties, lack of equipment or inadequate, lack of space, any difficulties and supply chain, volume of NPA sample, any severe side effect seen due to NPA. If yes how it was managed?*

# *Assistance from parents/colleagues during NPA procedure*

# *In terms of staff workload (time it takes) and skills*

# *In terms of parents’ understanding / attitude towards NPA procedure and referral process if required*

# *Overall attitude, motivation towards NPA*

# *Effect of COVID pandemic, ETC*

# Can you share your experience and viewpoint on whether or not NPA contributed to improving the identification of presumptive cases and diagnosis of TB among children in this health facility?

# *Probe for example for:*

# *Did it (and how did it) improve sample collection for testing?*

# *Did it (and how did it) improve/earlier diagnosis of TB in children, ETC…*

# What would you suggest to improve NPA in children for TB in this facility?

# Would you recommend NPA for routine use in your facility? And WHY?

# Stool sample collection

# Can you share your experience of implementing stool collection as part of TB Speed project? What is working well and what is not working well? And WHY so?

# *Probe for example for:*

# *Personal involvement in stool collection*

# *Description of procedures and what they were told and what they told parents*

# *Staff workload and skills?*

# *Any technical difficulties, lack of equipment?*

# *Difficulties to receive stool samples from children?*

# *In terms of parents’ understanding / attitude towards NPA procedure and referral process if required*

# *Overall attitude, motivation towards stool sampling*

# *Effect of COVID pandemic, ETC*

# Can you share your experience whether or not stool collection contributed to improving the identification of presumptive cases and diagnosis of TB among children in this health facility?

# *Probe for example for:*

# *Did it (and how did it) improve sample collection for testing?*

# *Did it (and how did it) improved/earlier diagnosis of TB in children, ETC…*

# What would you suggest to improve stool collection in children for TB in this facility?

# Would you recommend stool collection for routine use in your facility? And WHY?

# Xpert Ultra testing

# Is Xpert Ultra testing performed in this facility?

# *If yes performed, then continue with questions below*

# *If not performed: any specific reasons? No training? No equipment? reluctance? ETC…*

# Can you share your *experience of/perception of* performing Xpert Ultra testing in this facility as part of TB Speed project? What do you see as positive/not so positive? And WHY so?

# *Probe for example for:*

# *Knowledge of the procedure, personal involvement in performing Ultra*

# *Confidence in own technical skills, in relevance of the procedure*

# *Attitude, motivation*

# *Role of training/clinical mentoring*

# *Any technical difficulties- lack of equipment, sample volume, availability of cartridges, timely supply of cartridges, module failure, timely repair of module, electricity supply, electricity backup, error in results, invalid results*

# *Effect of COVID pandemic- any Xpert machines used to test for Corona virus, ETC*

# Can you share your *experience of/perception of* Xpert Ultra testing as *contributing* to improve the identification of presumptive cases and diagnosis of TB among children in this health facility?

# *Probe for example for:*

# *Improved trust in TB diagnosis*

# *Early and improved detection of TB and drug resistant TB, ETC…*

# Would you recommend Xpert Ultra for routine use in your facility? And WHY?

# What would you suggest to improve Xpert Ultra testing for TB in this facility?

# What is your *experiences/perception* of parent(s)/guardian(s) perception and acceptance of Xpert Ultra in this facility?

# Chest X-Ray (CXR) performance/use/reading

# Is CXR performed for the diagnosis of childhood TB in this facility?

# *If yes performed, then continue with questions below*

# *If not performed: any specific reasons? No training? No equipment? reluctance? ETC…*

# Can you share your *experience of/perception of* performing/using/reading CXR in this facility as part of TB Speed project? What do you see as positive/not so positive? And WHY so?

# *Probe for example for:*

# *Since when is there a CXR machine in the facility? Who does/ uses/reads CXR?*

# *Knowledge of role of CXR, personal experience in taking/using/reading CXR*

# *Confidence in own technical skills, in relevance of CXR*

# *Attitude, motivation*

# *Any technical difficulties- lack of equipment, availability of DR plates, availability of X-Ray reader and re-reader, electricity supply, electricity backup*

# *Effect of COVID pandemic, ETC*

# Can you share your *experience of/perception of* CXR as *contributing* to improve the identification of presumptive cases and diagnosis of TB among children in this health facility?

# *Probe for example for:*

# *Improved trust in TB diagnosis*

# *Improved diagnosis of TB, ETC…*

# Would you recommend CXR as routine service in your facility? And WHY?

# What would you suggest to improve taking/using/reading CXR in children for TB in this facility?

# What are your *experiences/views* about parent(s)/guardian(s) perception and acceptance of CXR in this facility?

# Clinical evaluation

# Can you share your experience of clinical evaluation as part of TB Speed project? What is working well and what is not working well? And WHY so?

# *Probe for example for:*

# *Personal experience with clinical evaluation*

# *Confidence in own clinical skills, discussion with colleagues*

# *Attitude, motivation*

# *Change in clinical practices /understanding of childhood TB compared to before TB-Speed?*

# *Effect of COVID pandemic, ETC*

# Can you share your *experience of/perception of* clinical evaluation as *contributing* to improve the identification of presumptive cases and diagnosis of TB among children in this health facility?

# *Probe for example for:*

# *Improved trust in TB diagnosis*

# *Improved diagnosis of TB, ETC…*

# Would you recommend that clinical evaluation is done routinely in your facility? And WHY?

# What would you suggest to improve clinical evaluation on children for TB in this facility?

# Clinical mentoring

# Can you share your experience about clinical mentoring in this health facility as part of TB Speed project? What is working well and what is not working well? And WHY so?

# *Probe for example for:*

# *Role as mentee and/or mentor? Clinical mentoring outside of TB-Speed?*

# *Attitudes towards clinical mentoring, acceptability or reluctance, and WHY*

# *Any difficulties while conducting/receiving clinical mentoring in this facility*

# *HCW workload, lack of time*

# *Effect of COVID pandemic*

# Can you share your experience whether or not clinical mentoring contributed to improving the skills of HCWs for the management TB and childhood TB in this health facility?

# *Probe for example for:*

# *Increased confidence/trust? Which improved skills in particular?*

# *Differences per mentees? Mentors? What key characteristics are important? Why?*

# *Better diagnosis and better quality of care for children, ETC…*

# Would you recommend clinical mentoring routinely in your facility? And WHY?

# What would you suggest to improve clinical mentoring in this facility?

# Decentralisation

# Can you share your experience about overall childhood TB diagnosis (and its different components) done at PHC-level? At DH-level?

# *Probe for example for:*

# *Before and during TB Speed project*

# *Impact of TB Speed training, support supervision*

# What worked well / not work well for childhood TB diagnosis at PHC-level? At DH-level?

# *Probe for example for:*

# *HCW workload, equipment*

# *Detailed objectives and guidelines, involvement from the head of facility/director*

# *Attitudes from HCWs not willing to be involved in aspects of diagnosis*

# *Challenges with the referral process: for HCWs (poor communication with the DH…) or for parents/guardians (transportation/distance/time/costs...), ETC.*

# *Acceptance and readiness of parents, motivations of parents for referrals*

# Would you recommend for the decentralization of childhood TB diagnosis interventions at PHC level/ in DH level? And WHY?

# Overall improvements

# To conclude our interview, what would be your suggestions based on your experience for improving the identification of presumptive cases and diagnosis of TB among children and the management of children with TB symptoms in this health facility? In the district overall?
