# Supplementary material for: Acceptability of decentralizing childhood tuberculosis diagnosis in low-income countries with high tuberculosis incidence: Experiences and perceptions from health care workers in Sub-Saharan Africa and South-East Asia
Source: PLOS Glob Public Health. 2023 Oct 11;3(10):e0001525. doi: 10.1371/journal.pgph.0001525 (PMC10566691; doi:10.1371/journal.pgph.0001525)
Supplement: Attachment — Submitted filename: PGPH_Joshi_Accept_Response_V1.0_2023-03-29.docx [file pgph.0001525.s002.docx]

**Dear Editor**

We would like to thank the 2 reviewers for their thorough review of the paper and suggestions for clarifications, improvements, further contextualization and interpretation of findings. You will find below are point-by-point responses to the comments and changes that have been made in the original manuscript in track changes.

**Reviewer 1:**

This is an interesting, thorough, and relevant paper. I just have a few comments and questions.

1. This study was carried out during the acute phase of the COVID-19 pandemic when TB testing in many places was down because people were reluctant to bring their child to a healthcare facility and some healthcare workers were reluctant to obtain sputum. Although the pandemic is mentioned a couple of times, it would be useful to include something in the discussion acknowledging the ways in which the conclusions from this paper might or might not be relevant going forward as things return to normal.

Response: We thank the reviewer for their comments and suggestions regarding the potential influence of Covid-19 on our study findings.

First, as a word of background, the Covid-19 pandemic directly affected the TB-Speed Decentralization Study itself, delaying the start date of the intervention phase and interrupting the study for 3 months in 2020, during the lead-in phase of the study. Once the intervention phase started, a specific Covid-19 taskforce was put in place in the TB-Speed project overall, with international, national and local key stakeholders, in order to support the sustained implementation of childhood TB diagnosis services in the study districts. “Extra-ordinary” conditions thus prevailed in these districts for a few months (regular, though distance, support and supervision of staff; provision of PPE stocks when other facilities may not have had them, contributed to address the challenges reported by health facilities). Also, several HCWs in the field as well as research team members were at some point infected with SARS-Cov-2.

Although we did not put in place a specific sub-study to investigate and describe in detail the consequences of Covid-19 on the childhood TB diagnosis package delivery, or its acceptance, there is little doubt that the pandemic affected overall access to care (lockdown and limited transport means), care seeking behaviors (fear of infection), as well as quality of care provided (certain facilities became “Covid centers” for a while, with limited general care activity). However, it should be noted that 1) the burden of Covid-19 lockdown and impact on healthcare services varied widely from one country to the other, from almost no changes and only restrictions to move from/to the distant capital city in Côte d’Ivoire to complete blockade and disruption in hospital care in Cambodia, and that 2) we conducted interviews between July and September 2021, at a time when most activities had returned to a normal/sub-normal level.

The HCWs interviewed in this study presented here did report COVID-19 specific challenges in their day to day activities and specifically in their delivery of childhood TB diagnosis services. We can assume that certain barriers and challenges such as transport challenges due to COVID-19 restrictions, would not be reported in non-Covid contexts and times. But we can assume that most perceptions and experiences of the childhood TB diagnosis package, such as the feeling of effectively improving diagnosis, or the burden of perceived additional workload, would remain relevant regardless of COVID-19.

We agree that it’s important to further acknowledge COVID-19 as a possible limitation to our study findings, and have amended the discussion lines 514-16 of manuscript (Track change version) as follows: -

**“***Although this study was conducted in different COVID-19 contexts, i.e. during and after COVID-19 measures,* we cannot rule out the fact that our findings may have been different in non-Covid times*.***”**

2. Were there any differences in results by country?

Response: We conducted both country-stratified analysis and overall analysis, and we did not identify striking differences in acceptability findings across countries. However, as we did not aim for a formal comparison to highlight similarities and differences between countries, we may have missed interesting differences.

HCWs overall reported positive perceptions of this diagnosis approach, despite large differences in their background, roles and responsibilities, and despite large differences in contexts (such as pre-existing level of equipment available, or electricity coverage) and some differences in implementation (support supervision varied between all countries, depending on national staff availability, accessibility etc.). That said, some acceptability findings were more strongly reported in some countries than others; of note these may reflect true differences in HCW experience, but may also be related to SSRA differential line of questioning. For example:

- HCWs in Mozambique from DH-focused district were extremely demotivated by the fact that they could not implement the full package.
- HCWs from Cameroon and Uganda found it challenging to take consent from some older children coming alone without parents to the health facility, which was not shared by HCWs of other countries.

We do not believe that these findings from one or two countries should be formally stated in the paper but we would be happy to hear the reviewer’s and editor’s view on this.

We have amended the paper as follows: 500-04.

*“And finally, the fact that we conducted a multi-country assessment of acceptability, highlighting common features across countries and not formally comparing countries, may have led to some over-simplification of findings, some being more relevant to certain settings than others and also maybe missing on interesting differences.”*

3. Given that some patients were referred from the PHC to the DH, it would be good to provide some context around how far these services were from each other? How logistically difficult and time-consuming was it for parents/caregivers to do this?

Response: Distance between the DH and the PHCs participating in this study largely varied, ranging from 4.5 km to 85.6km with large in-country variations, e.g. between 4.5 km to 35 km in Cambodia, 5 km to 85.6 km in Cameroon, or 15 km to 57 km in Uganda. We did not however document the precise distance between people’s homes and each facility, let alone between people’s homes and each type of facility. As discussed in the paper, we are aware that for parents/caregivers, accessing DH took time, had a cost, and was sometimes further challenged by poor availability of transport (worse during COVID-19 restrictions). These difficulties among parents were also experienced as a burden by HCWs, who felt that they were failing to reach the expected referral rates (and thereby not providing adequate care).

In order to be more specific about the distance challenges, we have added following sentences in lines 477-78 of manuscript as follows:

*“In the study, distance from PHCs to DH ranged between 4.5 km and 86 km.”*

4. On a similar note, part of the point of decentralizing services is so that patients do not have to travel as far – in these settings, what were these distance savings like? How far did they have to go for centralized services as compared with decentralized services?

Response: As explained in our introduction, most tuberculosis diagnostic tools and interventions, either already recommended by WHO or being evaluated, are currently concentrated at tertiary care levels. The TB-Speed Decentralization study evaluated 2 decentralization strategies. In districts implementing the DH-focused strategy, diagnosis of presumptive cases (either referred from PHC or screened at DH) was done at DH. In PHC-focused district, the decentralization of services was taken one step further, with both screening and diagnosis being done in PHCs (as well as DHs) except for CXR for which children were referred to DH if needed. So, both strategies brought services closer to patients. We did not collect data on the exact distance between patient’s residence and the DH or local PHC, thus we are unfortunately unable to compute the distance saved for parents in one strategy compared to the other.

5. How do these results generalize to settings where resources like digital chest x-ray do not exist at the decentralized service facilities? Are the PHCs and DHs included in this study comparable to some more rural and poorly resourced settings in high TB burden countries?

Response: Prior the start of the TB-Speed Decentralization study, we conducted a baseline assessment survey in the six study countries to select districts and health facilities. Selection criteria for district were: 1) rural or semi-urban district, 2) have one DH and 4 PHC at least that could participate in the study; 3) have functional X-ray services or capacity to perform X-ray at DH; 4) minimum human resource of a clinician, a nurse and a laboratory technician at PHC level; and 5) not receiving support from NGOs or other institutions except if in line with national recommendations as per NTP guidelines. Furthermore, tuberculosis case notification and population size of districts were considered in the selection of the districts.

Of the 179 PHCs in 32 districts from 5 countries (Cambodia, Cameroon, Cote d’Ivoire, Mozambique and Uganda), 75% of PHCs had specific staffs for TB management and only 30% of these staffs were trained for pediatric TB in these PHCs. 54% of PHCs referred children to higher health care level for TB diagnosis. Although around 70% of PHCs collected microbiological sample on site, only 7% of them collected samples from children. Around 46% of PHCs had onsite laboratory with 3.3% having X-pert MTB/RIF in the laboratory.

We selected 12 DHs and 47 PHCs based on the results from this baseline assessment. Out of the 47 PHCs, 34% were tuberculosis diagnosis and treatment units. Overall, we thus can assume that the sites participating in the study were representative of largely rural and poorly resources facilities in TB-burden countries.

Regarding the CXR service delivery. In the study, the decentralization strategies tested included CXR performed at DH only. Among the 12 DH, 10 had analog radiography available prior to the study. CXR DR plates were set-up in all 12 DH. CXR reading and interpretation was done at DH in the DH-focused districts and at PHC-level for the PHC-focused districts (following specific CXR training sessions, clinical mentoring throughout the intervention phase).

We added following sentence in discussion section of manuscript in between line 509-12.

*“Prior the start of the TB-Speed decentralization study, we conducted a baseline assessment survey in the six study countries to select districts and health facilities, and the 59 facilities included are representative of largely rural and poorly resources facilities in TB burden countries.”*

**Reviewer 2:**

**General Comments**

In this large, qualitative study, the investigators conducted key informant interviews with 130 healthcare workers participating in the TB-Speed Decentralization study, a pre-post implementation study of a multi-component pediatric TB diagnostic package in 6 countries in sub-Saharan Africa and Southeast Asia. The goals of this sub-study were to characterize the acceptability to healthcare workers of decentralizing TB diagnostic evaluation to primary health centres and district hospitals, including introducing systematic TB screening, collection of nasopharyngeal aspirates and stool specimens and molecular testing with GeneXpert MTB/RIF Ultra, as well as enhanced clinical evaluation, sometimes with radiographic evaluation and referral to district hospitals. After carrying out thematic analysis and deductive coding using the Theoretical Framework of Acceptability, the authors show that the package was broadly acceptable.

**Major Comments**

This is a well-conceived, and generally well-executed study that offers valuable context on health worker perceptions of a broad package of pediatric TB diagnostics implemented in real-world settings in multiple high-burden countries. I have a few questions for the authors on the reporting and interpretation of the study

1. It would be useful to have more information on the setting and context of the parent study, in particular a) what was the general pre-intervention standard of care for pediatric TB evaluation at these sites, and b) how were the different decentralization packages implemented in the different countries. Table 1 is helpful on the different intervention components, but it’s not quite clear where or why different models (i.e. PHC-focused versus DH-focused) are being used, and who is providing which services where. This need not be a comprehensive description; in fact, just a short overarching explanation so readers don’t necessarily have to study Table 1 to understand the intervention. Is it accurate to say that in the DH-focused strategy, only symptom screening is decentralized, while in the PHC-focused strategy, screening and diagnosis are decentralized and DH referral is only necessary for chest radiography?

Response: As stated by the reviewer in their comment, in each country, the 2 districts were randomly allocated to either the DH-focused or PHC-focused decentralization models. In DH-focused districts, the full diagnostic assessment of children with presumptive tuberculosis (either referred from PHC or screened at DH) was done at DH, hence the role of PHC was to identify children with presumptive tuberculosis through systematic screening and to refer them to DH. In PHC-focused districts, both screening and full diagnostic assessment and decision were done at PHCs (as well as DHs) except for CXR for which children were referred to DH, when CXR was indicated.

a) Due to the study design, we were unable to formally document details of the standard of care during the observation phase, hence to document changes in clinical practices. Overall, none of the PHCs implemented children-adapted sample collection methods and molecular testing. In Uganda and Mozambique, pediatric TB services were already partially decentralized but only had access to smear microscopy performed on expectorated sputum for microbiological diagnosis. We have tried to explain this further, amending the Study design and setting paragraphs (lines 138-42), as follows:

“*In Uganda and Mozambique, tuberculosis diagnosis services were partly decentralized at DH and even at PHC-level; this consisted mainly in smear microscopy performed on expectorated sputum for microbiological diagnosis, and did not include NPA and stool sample collection and sample testing using Xpert Ultra.”*

b) Regarding how decentralization packages were implemented in different countries, first an overall standardized implementation guide was co-constructed with all countries, and then both country and locally-adapted guides were defined and put in place; this means slight differences in implementation processes in each country. For example, in Sierra Leone instead of just referring presumptive children to DH, some PHCs in the DH-focused district provided stool containers to patients and explained them to collect stool and transport to DH lab on the same day of visit to minimize diagnosis delay; in Côte d’Ivoire, some HCWs from PHCs implemented a “supportive referral system”, sometimes themselves escorting children with their parents to DH.

Table 1 was revised following your remarks. The following sentence was added in the study design and setting section of the manuscript, line 157 and 158.

*“Interventions were implemented based on international and country specific implementation guide, including local adaptations.”*

2. Building on #1, the Results subheadings summarize the key components of the intervention and effectively link the acceptability findings to the intervention components. In addition to better describing who is doing these steps, would suggest using parallel terminology and ordering of the Table 1 and Results headings (currently they are only loosely aligned).

Response: We thank the reviewer for their suggestion that indeed will contribute to better explain the TB-Speed intervention components, decentralization models and settings. Table 1 was further revised as suggested.

3. In the Methods, under study population, could you explain which axes of diversity guided your purposive sampling? Gender is mentioned, but it is not clear if health workers were sampled by profession or by the role that they played in offering pediatric TB services? If the later, it would help to describe this distribution in Table 2. Could you also comment in the Methods or Results as appropriate on the distribution of health worker professions by country (e.g., in Cambodia and Uganda, a much broader cross-section of healthcare workers participated). This would better set the stage for understanding the limitation you mention that doctors and other clinicians were not included in some countries even though it seems that you had planned to interview them.

Response: We thank the reviewer for their suggestion. Purposive sampling of HCWs was based on their role in delivering components of the childhood TB diagnosis package. We had initially planned to ensure gender balance but this criteria was too constraining, and not as important as ensuring proper selection based on HCW position/professional role. We added this clarification in the Study population section (Page 161-65) of the manuscript as below.

*“A purposive sample of 1 to 5 HCWs from each participating health facility was recruited for interviews. HCWs were selected considering the total staff number in each facility, and based on their* *experience in delivering components of the childhood TB diagnosis package within their health facility (screening, clinical evaluation, sample collection, sample testing, performing or reading chest X-Ray).”*

As suggested by the reviewer, we revised the section on the distribution of HCWs professions by country in the results section, as follows (line 212 – 216):

*“Globally, more than half of the HCWs interviewed were nurses (ranging from 31% in Uganda to 87% in Côte d’Ivoire) followed by clinical officers/medical assistants (18%) and medical doctors (10%). The highest number of medical doctors interviewed were from Cameroon (21%) and none of the medical doctors were from Sierra Leone.”*

4. Appendix 1 shows and interview guide with 36 questions, a very large number for a single sitting. Was the instrument piloted, how were the interviewers trained to ask the questions, and what was the duration of the interviews? Were the interviews completed in a single session?

Response: We agree with the reviewer that the interview guide was long indeed. It was meant to be as comprehensive as possible, listing key questions and probes for each of the intervention components. SSRAs were trained to adapt their interview conduct and line of questioning according to the profile of the respondent and her/his role in childhood TB diagnosis (investigating either experience or perceptions or both). We conducted several role-play sessions with all SSRAs prior fieldwork; they also had been involved in pre-intervention qualitative data collection and were familiar with this approach of adapting the guide to the respondent’s profile. That said, childhood TB diagnosis is complex, the intervention tested is multi-faceted, and many HCWs are very talkative and like talking about their work, so the duration of interviews often ranged between 1 hour to 1 hour and 30 minutes. Interviews were completed in single session, though discussions were sometimes interrupted by patients or HCWs requiring the respondent’s input.

5. Did the authors assess for thematic saturation?

Response: SSRAs were instructed to interview between 1 and 5 HCWs per facility, as per protocol; they were also advised to recruit additional staff if they believed it would provide interesting insight and additional quality data (i.e. purposively recruiting laboratory technicians or radiographers in the facilities that had such staff). However, we did not formally assess data saturation during data collection.

We amended the manuscript and added following sentence under study population (line 167-72)

*“SSRAs were advised to recruit additional staff if they believed it would provide interesting insight and additional quality data (i.e. purposively recruiting laboratory technicians or radiographers in the facilities that had such staff).”*

And also amended in limitation section of manuscript which reads as follows (line 493-95)

*“As a consequence, we may have missed some specific insight from key actors, and we cannot ensure that we reached data saturation.”*

6. There is only loose alignment between the findings in Table 4 and the summary of the multi-level factors in the Discussion session. For example, under the individual factors, I don't find the particular notion of ethics discussed in the Results or the Tables (where ethics is discussed in terms of patient acceptance and discomfort). Moreover, there is a discussion about PHC-focused HCW being more engaged that DH-focused strata, but most of if not all of the quotes in the Results and in Table 3 show a high level of engagement in DH-focused facilities. In the Discussion of facility or health system level factors, the factors that were acceptable (or facilitated acceptability) are not mentioned.

Response: We thank the reviewer for their helpful comment, contributing to a better alignment between findings, discussion points and the supporting tables.

Among the individual-level factors contributing to the acceptability of decentralizing diagnosis, ethicality is key. Ethicality is defined by Sekhon et al. as the “extent to which the intervention has good fit with individual’s value system”. In our study, HCWs explained that decentralizing childhood TB services meant bringing services closer to patients i.e. improved access to care and improved service coverage: here, we considered that HCWs were expressing how “ethical” the intervention was to them. This is reflected indeed in table 4. In Table 3 we opted for presenting quotes that relate to another aspect of ethicality, which we may summarize as “first do no harm” (preventing discomfort etc..).

Self-efficacy was another important component of acceptability as reported by HCWs. This is what we aimed to explain in the discussion section, when we refer to sometimes different levels of engagement in the PHC-focused and DH-focused districts/facilities: in PHC-focused district, staff at PHC-level screened children and were then responsible for initiating the next steps of diagnosis, thereby feeling empowered; in DH-focused facilities, some regretted having to refer children straight after screening, for further diagnosis at DH, i.e. not being able to take part in the diagnosis process per se.

Regarding the balance of quotes between DH/PHC and DH-focused/PHC focused settings. To preserve “anonymity” of DH sites from DH-focused districts, we did not reference their quotes by specifying the type of decentralization strategy (which would equate to naming the DH), but we specified country, type of district and position of HCWs in their health facility for the management of childhood TB.

As per the reviewer’s suggestions, the discussion section and table 4 were revised in order to better align concepts discussed and words used.

In Paragraph 6 (line 472-75) under structural level factors-

*“This was further challenged when children were accompanied by grand parents and siblings, who were not always well-informed about the child’s health status and did not have parental authority to allow further TB diagnosis procedures.”*

In the last paragraph (line 512-14)

*“Together with the large sample size, implementation of interventions in different sites and with different cadres of HCWs contributes to the generalizability of our findings.”*

Table 4 was also revised to better align with the discussion.

**Minor Comments:**

1. Line 55: Consider specifying "stool collection" to make the reference to “cultural practices” a little clearer

Response: We thank the reviewer for their suggestion and modified the manuscript accordingly.

2. Line 337: Could you explain the meaning of the word collaborators? Who are they describing? Is the speaker suggesting that these individuals accompany families all the way from the PHC to the DH?

Response: In Côte d’Ivoire, in the context of our study, “collaborator” means “colleague”. The HCW here is indeed describing a situation in which PHC staff may end up accompanying a presumptive (TB) child referred from PHC along with parents, and make sure he/she reaches the DH and is seen by a staff trained to TB-Speed procedures. This is not the usual referral process within the NTP or the TB-Speed project; this was implemented upon the individual initiative of certain facilities/HCWs (showing high motivation to make sure referred children reach the DH and receive the full diagnosis package). We have further clarified in the manuscript (line 350) what was meant by “collaborator” as follows:

*“At the general hospital there are crowds of people, so always we have them (parents) accompanied by one of our collaborators (colleague)…*

3. Line 424: Suggest changing "was shown" (which implies "in this study") to "has been shown in other contexts", or similar.

Response: We revised the manuscript as suggested. (Now line 436)

4. Line 480: Could you elaborate on why you think SSRAs may not have uncovered all the relevant themes? One might assume the opposite, if SSRAs from the same country are better able to recognize and probe on local nuances.

Response: There are indeed pros and cons when conducting multi-country qualitative research based on a common interview guide. We agree with the reviewer’s assumption and we have added the following sentence in the last paragraph (line 522-23) of the discussion.

*“Local SSRAs would have been able to recognize and better probe the country specific problems during interviews.”*

5. Line 481, in the same vein as the previous comments, a diversity of sites and perspectives would not be a weakness, but a strength from a qualitative methods perspective.

Response: We thank the reviewer for their comment. We agree that there are also pros and cons in diversity. In line 481 we reported as a limitation the fact of *representing common features* across countries which may have led to oversimplification of the findings; but we also agree with you that diversity of sites and respondents is a strength from a qualitative research’s perspective (allowing to investigate different contexts and mechanisms), which we have explained in last paragraph of discussion (line 507-09).

*“The multi-country design of our study also constitutes one of the major strengths of this work. Indeed, it provides insight into the perceptions and experiences of HCWs working in different health care systems, different socioeconomic and cultural contexts.”*

6. Table 1: “Greater than”, or “greater than or equal to” 2 weeks? “Documented” or “Reported” weight loss? D7=Day 7? After presentation? After treatment initiation?

Response: The recommendation was to assess cough or fever for more than 2 weeks, hence >2 weeks. During screening at triage, we used both documented and reported (if documented was not possible) weight loss so we added reported as well in table 1. D7=Day 7 (after presentation) indeed – we revised the manuscript to further clarify this.

7. Line 509: “every sense of the word” might be better stated – the authors found that the strategy was broadly acceptable across all 7 domains of the Theoretical Framework of Acceptability, but also (as said at the opening of the Discussion) elements that were not acceptable (or were barriers to acceptability). Similarly, the statements about sustainability or scalability seem out of scope in that they imply that strategies to promote acceptability would also enhance sustainability or scalability.

Response: We thank the reviewer for their suggestion. We have revised the conclusion paragraph (line 534-41) as follows.

*“In this multi-country qualitative study, HCWs at DH- and PHC-levels perceived and experienced decentralized childhood tuberculosis diagnosis as acceptable. Among others, they understood its purpose, its fit with their personal and professional values, and they were overall very motivated to provide what they perceived as improved quality of care. Acceptability however seemed to be hampered by several operational and feasibility issues, resulting in feelings of frustration or burden sometimes. Innovative referral mechanisms for patients, samples and chest X-ray could improve the acceptability of decentralized interventions for diagnosing childhood tuberculosis.”*
