# Supplementary material for: Acceptability of decentralizing childhood tuberculosis diagnosis in low-income countries with high tuberculosis incidence: Experiences and perceptions from health care workers in Sub-Saharan Africa and South-East Asia
Source: PLOS Glob Public Health. 2023 Oct 11;3(10):e0001525. doi: 10.1371/journal.pgph.0001525 (PMC10566691; doi:10.1371/journal.pgph.0001525)
Supplement: Attachment — Submitted filename: PGPH_Accept_Response_V2.0_2023_07_05.docx [file pgph.0001525.s003.docx]

Dear Editor

You will find below are point-by-point responses to the second round of comments by reviewers and changes that have been made in the original manuscript in track changes.

**Reviewer 1:**

1. **Comment-** Address specific ways in which conclusions may or may not be relevant to post-covid times.

Response: We have amended the discussion section (track change version from the previously revised paper) to be more explicit in the paper about the potential influence of COVID-19 on our study findings. Lines 540-48 now read as follows:

“Although this study was conducted in different COVID-19 contexts, i.e. during and after COVID-19 measures, we cannot rule out the fact that our findings may have been different in non-Covid times. *For example, certain barriers and challenges such as transport challenges due to COVID-19 restrictions, would not have been reported in non-COVID contexts and times. On the other hand, we may hypothesize that most perceptions and experiences among HCWs of the childhood TB diagnosis package, such as the feeling of being able to improving diagnosis, or the burden of perceived additional workload, would remain relevant regardless of COVID-19.***”**

2. **Comment-** On a similar note, part of the point of decentralizing services is so that patients do not have to travel as far – in these settings, what were these distance savings like? How far did they have to go for centralized services as compared with decentralized services?

Response: We have addressed this issue of increase access and distance saving by adding the following sentence in discussion section of manuscript, lines 503-06.

*“Overall across the 6 countries, distances from PHCs to DH ranged between 4.5 km and 86 km. Although it is likely that the decentralization of services at PHC-level reduced distance barriers for several patients, we were unable to formally compute the distance saved.”*

3. **Comment-** Address with further details in manuscript.

**Previous comment (no. 5)**- How do these results generalize to settings where resources like digital chest x-ray do not exist at the decentralized service facilities? Are the PHCs and DHs included in this study comparable to some more rural and poorly resourced settings in high TB burden countries?

Response: We have revised the study setting section by adding information on the facility characteristics, between lines 145-59, as follows:

*“**Prior to the start of the TB-Speed decentralization study, we conducted a baseline assessment survey in the six study countries to select districts and health facilities. Of the 179 PHCs surveyed in 32 districts from 5 countries (Cambodia, Cameroon, Cote d’Ivoire, Mozambique and Uganda), 75% of PHCs had specific staff members for TB management and only 30% were trained for pediatric TB. Although around 70% of PHCs collected microbiological sample on site, only 7% of them collected samples from children. Around 46% of PHCs had onsite laboratory with 3.3% having X-pert MTB/RIF in the laboratory. From this survey, districts for the study were selected with National TB Programs (NTPs) with the following criteria: 1) rural or semi-urban, 2) one DH and 4 PHC at least that could participate in the study; 3) have functional X-ray services or capacity to perform X-ray at DH; 4) minimum human resource of a clinician, a nurse and a laboratory technician at PHC level; 5) not receiving support from NGOs or other institutions except if in line with NTP guidelines; this selection also took in consideration the tuberculosis case notification and population size of districts. Overall 12 DHs and 47 PHCs were selected including, 34% that were tuberculosis diagnosis and treatment units..”*

And we added one sentence in discussion section of manuscript in between line 535-38.

*“…… different socioeconomic and cultural contexts, while representative of largely rural and poorly resources facilities in TB burden countries. Feasibility and acceptability of introducing innovative technologies in such districts is therefore representative of challenges that would be faced in most districts in project countries.”*

**Reviewer 2:**

1. **Comment (no. 1b).** How were the different decentralization packages implemented in the different countries. Table 1 is helpful on the different intervention components, but it’s not quite clear where or why different models (i.e. PHC-focused versus DH-focused) are being used, and who is providing which services where. This need not be a comprehensive description; in fact, just a short overarching explanation so readers don’t necessarily have to study Table 1 to understand the intervention. Is it accurate to say that in the DH-focused strategy, only symptom screening is decentralized, while in the PHC-focused strategy, screening and diagnosis are decentralized and DH referral is only necessary for chest radiography?

Response: Regarding how decentralization packages implemented in different countries, in this second round revision, we further revised the study design and settings section of the manuscript between line 159-77 (changes made is indicated in red and italics in the paragraph below) to make it clearer. This paragraph now reads as follows:

*“*Two levels of decentralization were implemented in each country, *i.e. a DH-focused and a PHC-focused strategy, based on the hypothesis that decentralizing tuberculosis diagnosis down to the PHC level would improve access but could face additional feasibility challenges compared to DH, related to staff availability and capacity. The study was therefore designed to formally assess and compare the effect on tuberculosis case detection and implementation challenges of the two proposed strategies*. In DH-focused districts, the DH and 4 PHCs conducted systematic symptom-based screening for tuberculosis among children, using simple questions asked either at triage and/or in consultation rooms. Children identified as presumptive tuberculosis at PHC-level were referred to DH (parents did not receive transport reimbursement) for Xpert MTB-RIF Ultra (Ultra) testing on nasopharyngeal aspirate, stool and/or expectorated sputum samples, as well as strengthened clinical evaluation, digital chest X-Ray with a simplified reading approach, and final diagnosis of tuberculosis. Details of the activities conducted by HCWs are presented in table 1. In PHC-focused districts, the DH and 4 PHCs were equipped to implement the comprehensive childhood tuberculosis diagnosis package; only chest X-Ray was done at DH-level *for all children seen at DH and in* *those that were* still symptomatic after a week *at PHC* (parents received transport reimbursement); images were made available to clinicians in PHCs. Interventions were implemented based on an international and country specific *implementation guides with* local adaptations.”

2. **Comment-** Appendix 1 shows and interview guide with 36 questions, a very large number for a single sitting. Was the instrument piloted, how were the interviewers trained to ask the questions, and what was the duration of the interviews? Were the interviews completed in a single session?

Response: We have included more details about the data collection procedures, please see amendment to the methods section (line 198-201-03 and 203-05),

*“We piloted the interview guide in one country (Sierra Leone), during two interviews, and discussed the process within the multi-country team of SSRAs before finalizing a standard version of the guide, to be used in all six countries.”*

*“Interviews were completed in a single session, though they were sometimes interrupted by patients or other HCWs requiring the respondent’s input. The duration of interviews usually ranged between 1 hour and 1 hour and 30 minutes.”*

3. **Comment-** The authors say they can't "ensure saturation was reached", which is an odd statement. Typically interviews are stopped when the interviewers hear the same comments repeatedly. Why did the authors stop the interviews prior to this? Or do the authors simply mean that saturation was reached from the participants in question, but further insight may have been aqcuired had a more diverse group been recruited?

Response: To further answer this new comment from the reviewer, SSRAs were instructed per protocol to interview between 1 and 5 HCWs per facility. This minimum-maximum sample size was pragmatic, suggested on the basis of relevance (we targeted those who took part in some aspects of childhood TB diagnosis, and did not expect those to be less than one or more than 5, especially at PHC-level) and feasibility (we wished to ensure that the study enrolled a “manageable” number of respondents, that would allow for multi-site analyses; we also assumed that workload among HCWs would prevent high participation rates). SSRAs were not able to talk to all the HCWs that they had targeted due to their lack of availability. However they were encouraged to recruit additional staff if they believed it would provide interesting insight and additional quality data (i.e. purposively recruiting laboratory technicians or radiographers in the facilities that had such staff). However, we did not formally instruct SSRAs to aim for data saturation at country-level when recruiting participants. Thus the fact that this is mentioned as a study limitation that we have slightly amended following your recommendation between line 513 and 521, and now reads as follows.

“First, *our study sample was essentially pragmatic.* We were not able to include in our study population representatives of all key HCWs involved, in each country, in decentralizing the comprehensive childhood tuberculosis diagnosis approach. This was mainly the case among medical doctors and skilled clinicians, unavailable for study participation due to high workload or being absent onsite during data collection. As a consequence, we may have missed some specific insight from key actors. *Due to the multi-country, multi-site, and multi-HCW profiles, we did not aim for data saturation in each specific setting and population.”*
